# The cost of chronic strain: Sleep disruption, allostatic load, and the impact on adverse pregnancy outcomes

**DOI:** 10.1002/pmf2.70389

**Published:** 2026-08-10

**Authors:** Iona M. A. Palmer, Naima K. Chowdhury, Martha Gulati, Helen E. Collins, Clara G. Sears

**Affiliations:** ^1^ University of Louisville School of Medicine Louisville Kentucky USA; ^2^ Center for Cardiometabolic Science, Christina Lee Brown Envirome Institute, Division of Environmental Medicine, Department of Medicine University of Louisville Louisville Kentucky USA; ^3^ Christina Lee Brown Envirome Institute, Division of Environmental Medicine, Department of Medicine University of Louisville Louisville Kentucky USA; ^4^ Davis Women's Heart Center, Department of Cardiology Houston Methodist DeBakey Heart and Vascular Center Houston Texas USA

**Keywords:** heart, maternal morbidity, maternal mortality, stress

## 1 INTRODUCTION

It has long been recognized that pregnancy is associated with fatigue and poor, disrupted sleep. Research supports this understanding, indicating around 40% to 70% of pregnant women experience sleep disturbances [1, 2]. These disruptions are partly driven by the dramatic physiologic changes of pregnancy that place substantial strain on the maternal body. For example, hormonal fluctuations, nocturia, fetal movement, weight gain, and changes in respiratory physiology, all of which are critical components of a healthy pregnancy, can reduce sleep duration and worsen sleep quality [1]. Importantly, not all sleep disruptions are benign or driven entirely by “normal” physiological changes. More severe sleep disruptions may occur in situations when additional stressors impact sleep health, such as occupational demands, caregiving responsibilities, socioeconomic conditions, and underlying chronic health conditions.

Because sleep problems are often culturally normalized during pregnancy, they may be minimized by patients and overlooked by clinicians during routine clinical encounters. This is concerning because evidence suggests that, for some pregnant women, sleep disruptions are severe enough to meet the diagnostic criteria for a sleep disorder. Additionally, research suggests sleep disorders such as insomnia, restless leg syndrome, and obstructive sleep apnea (OSA) increase in prevalence during pregnancy [1]. Accumulating epidemiological evidence indicates that disrupted and disordered sleep during pregnancy is associated with a range of adverse maternal and neonatal outcomes, including hypertensive disorders of pregnancy (HDP), gestational diabetes mellitus (GDM), preterm birth, fetal growth restriction, perinatal mood disorders, and severe maternal morbidity [1, 3]. Notably, insomnia appears to confer risks independent of OSA and has been associated with preterm birth, placental disease, and delivery of small‐for‐gestational‐age infants [1]. Cardiometabolic adverse pregnancy outcomes(APOs), including gestational diabetes and HDPs, complicate up to 25% of all pregnancies in the United States, contributing a major share of maternal morbidity and death [4]. The danger of these gestational complications extends long after delivery, for experiencing an APO like HDP or GDM strongly predicts future maternal cardiometabolic disease. Moreover, it is important to recognize that these risks are not distributed equally, highlighting the central role of social determinants in influencing pregnancy outcomes [4, 5].

Some of the strongest evidence supporting a causal association between poor sleep and APOs comes from studies of pregnant individuals experiencing disrupted sleep due to shiftwork schedules. In these studies, rotating schedules, particularly those involving night shifts, are associated with higher odds of GDM, preeclampsia, and HDP as well as adverse fetal outcomes, including preterm birth and fetal growth restriction [6]. Beyond shiftwork, however, limited research has been conducted to untangle upstream causes and characteristics of sleep disruptions that meaningfully contribute to APO risk [6]. Furthermore, prior epidemiological studies in this area are often impacted by residual confounding and struggle to establish the temporality between sleep disruption and cardiometabolic dysfunction. As a result, the extent to which sleep disruptions cause APOs or, rather, act as an early symptom that perpetuates underlying pathology remains unclear. We believe the allostatic load framework offers a useful conceptual model to advance scientific understanding in this area and could inform targeted interventions aimed at reducing APO risk.

## 2 ALLOSTATIC LOAD AND PREGNANCY

**FIGURE 1:**
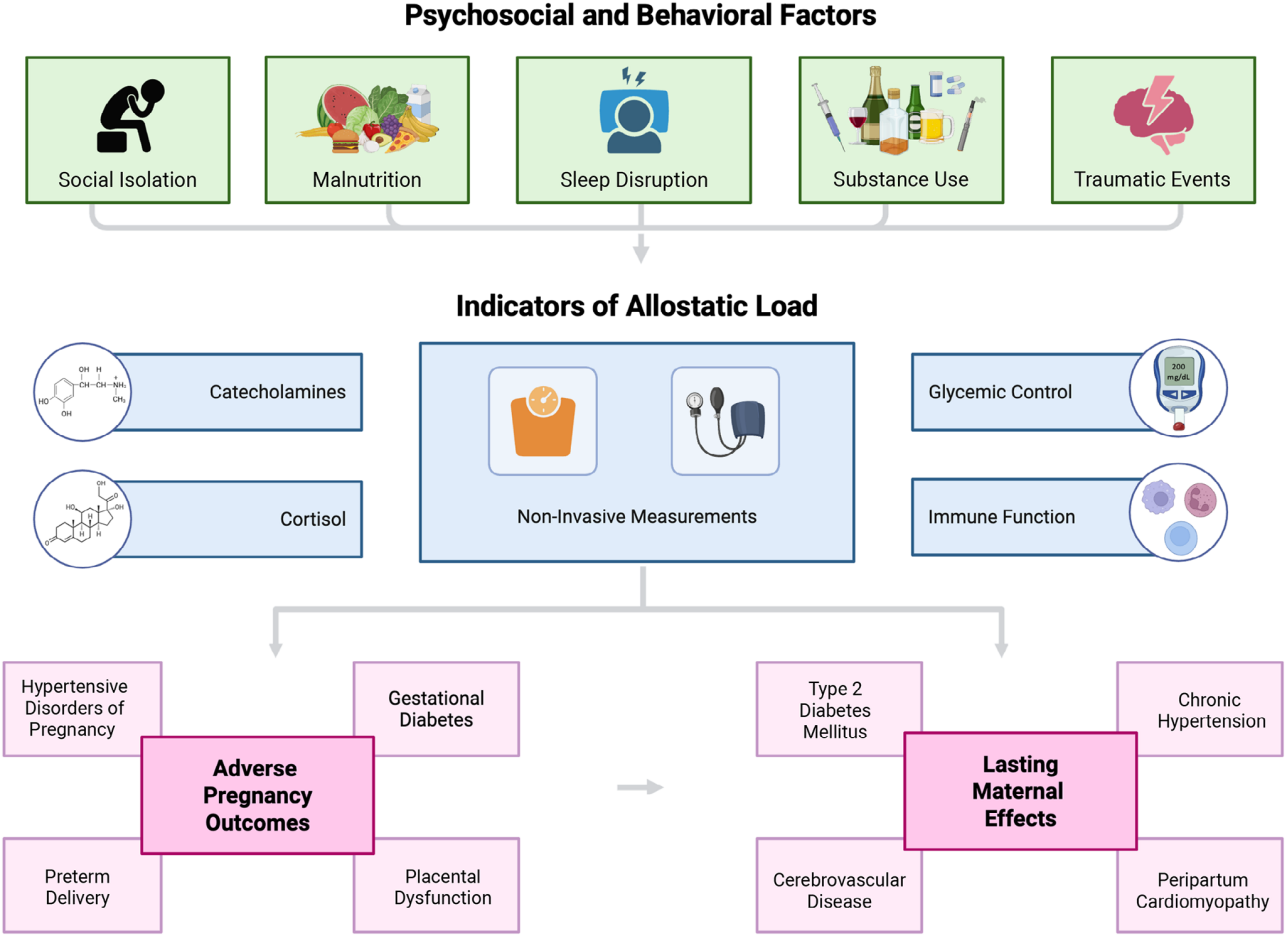
The impact of psychosocial stressors on adverse pregnancy outcomes. A variety of psychosocial factors—including social isolation, a history of traumatic experiences, substance use, inadequate nutrition, and sleep disruption—can increase biomarkers of allostatic load. These biomarkers include biochemical measures such as cortisol and catecholamines; blood‐based clinical indicators such as blood glucose, HbA1c, and immune cell populations; and noninvasive physical measures such as blood pressure and body mass index. Elevated allostatic load may predispose individuals to adverse pregnancy outcomes and, subsequently, long‐term maternal cardiometabolic consequences.

Allostatic load is conceptualized as the cumulative “wear and tear” that results from prolonged activation of stress‐response pathways due to repeated psychosocial, physical, and environmental challenges [7] (Figure 1). Measurement of allostatic load relies on a composite of biomarkers, which are classified as primary or secondary mediators [7]. Primary mediators, such as cortisol, cytokines, and catecholamines, reflect the physiological effects of an acute stress response. Secondary mediators, such as lipid profiles, waist‐to‐hip ratio, and blood pressure, reflect the longer‐term cardiometabolic effects of chronic stress exposure [4, 7]. Previous literature has revealed associations between elevated allostatic load and socioeconomic disadvantage, lower educational attainment, discrimination, and other chronic psychosocial stressors [7]. While there is not one “gold standard” method to derive the allostatic load metric, there appears to be some consensus that the composite measures provide a quantification of biological dysregulation that is indicative of future disease risk. Higher allostatic load has been associated with increased risk of adverse long‐term outcomes, particularly cardiovascular disease (CVD) and premature mortality [4, 7].

Recent obstetric research also suggests that higher allostatic load may be associated with increased risk for APOs [4, 5]. Retrospective studies have found that individuals who develop preeclampsia often exhibit markers of elevated allostatic load in early pregnancy, highlighting chronic physiologic dysregulation from repeated stressors as a key factor in the development of HDPs [4]. Prospective cohort studies further demonstrate that women with higher allostatic load in early pregnancy have approximately 50% higher odds of experiencing an APO compared with women who had lower allostatic load, with the strongest associations observed for HDPs [4]. Notably, the components most strongly associated with APOs—body mass index, systolic and diastolic blood pressure, triglycerides, insulin, and high‐sensitivity C‐reactive protein—are also well‐established biomarkers of increased CVD risk independent of pregnancy [4, 5].

## 3 DISRUPTED SLEEP AND ALLOSTATIC LOAD DURING PREGNANCY

The allostatic load framework has been leveraged to examine the adverse impacts of sleep disruption on health. When inadequate and poor‐quality sleep persistently activate both the sympathetic nervous system and the hypothalamic‐pituitary‐adrenal axis, these stress‐response systems can become dysregulated [4, 8]. Ultimately, these interconnected biological pathways promote widespread oxidative stress and inflammation, thereby increasing allostatic load [1, 9]. These same neuroendocrine, inflammatory, and cardiometabolic pathways that are affected by sleep and circadian disruption have been implicated in the development of HDP and GDM [1, 9].

Conversely, high allostatic load may further contribute to sleep disruption. Pregnancy essentially acts as a physiological “stress test” that requires substantial activation of allostatic processes to maintain stability while supporting the dramatic changes that occur across trimesters. This stress test may unmask underlying cardiometabolic vulnerabilities that went undetected prior to conception [8]. When the body struggles to adapt to the physiological demands of pregnancy, the neurobiological systems responsible for regulating sleep may also become disrupted. Therefore, individuals who enter the first trimester with elevated allostatic load due to chronic stressors may be at greater risk for developing sleep disturbances during pregnancy. Once sleep disruption occurs, it may further perpetuate underlying physiologic dysregulation, acting as a “second hit” that increases APO risk.

Recent epidemiological studies provide some evidence that sleep‐disordered breathing and poorer sleep quality during early pregnancy are correlated with higher allostatic load, although the direction of this relationship cannot be established in these studies [1, 2, 9]. Overall, this literature does suggest that elevated allostatic load accompanying disrupted sleep may compromise the gravid body's ability to meet the demands of gestation, and thereby increase risk for APOs.

## 4 ADDRESSING SLEEP DISRUPTIONS TO IMPROVE MATERNAL HEALTH

While some barriers to optimal sleep health are admittedly difficult to rectify, evidence suggests that existing nonpharmacological interventions can be adapted to improve sleep during specific stages of pregnancy. For example, physical activity interventions have been shown to improve sleep quality during pregnancy, potentially through reductions in inflammation and physical discomfort [10]. Clinicians could incorporate sleep health screening into preconception and prenatal visits to ensure that sleep disorders are diagnosed and treated as early as possible, optimizing maternal health. Additionally, clinicians can provide pregnant patients with education on healthy sleep behaviors, as well as resources regarding local, state, and federal protections, such as the Pregnant Workers Fairness Act (PWFA), which may help them work with their employer to reduce the negative impact of shiftwork. However, it remains unclear whether these types of interventions improve dimensions of sleep health beyond sleep quality, such as sleep duration, or whether improvements in sleep health subsequently reduce APO risk. Scientific advancement in this area has been limited, in part because pregnant women are often excluded from clinical trials evaluating interventions for poor sleep health. Nevertheless, advancing research in this area is critical.

Leveraging the allostatic load framework to better understand the biological mechanisms underlying problematic sleep disruption could facilitate the development of targeted interventions to improve sleep during pregnancy and reduce related APO risk. Meaningful advancements will require multidisciplinary collaborations among basic scientists, clinicians, and population health researchers to elucidate the underlying biological mechanisms at play across organ systems and the real‐world factors contributing to their disruption. Future research should continue to examine how biological, behavioral, and social factors interact to influence sleep health and allostatic load. It is critical to consider the role of social determinants of health in this area; these factors can shape the risk for sleep disorders during pregnancy, as well as directly increase risk for APOs. Particular attention should also be given to clarifying the temporal relationship between sleep disruption, physiologic dysregulation, and the development of conditions such as HDP and GDM, in addition to long‐term CVD risks.

Clinically, a holistic approach to maternal health—one that incorporates assessment of cardiometabolic risk, sleep health, and social determinants of health before, during, and after pregnancy—may improve identification of individuals at increased risk. By shifting focus from the treatment of complications after they arise to identifying and modifying underlying risk factors, clinicians may have an opportunity to improve both perinatal outcomes and long‐term cardiovascular health across the life course.

## CONFLICT OF INTEREST STATEMENT

M.G. reports serving on an advisory board for Amgen, Bayer, and Medtronic. She serves on a Data and Safety Monitoring Board for an investigational lipid‐lowering product for Merck. The other authors declare no conflicts of interest.
